# Ultrasound-driven ROS-scavenging nanobubbles for synergistic NASH treatment via FXR activation^☆^

**DOI:** 10.1016/j.ultsonch.2025.107352

**Published:** 2025-04-26

**Authors:** Jianru Lin, Jialin Chen, Mengdie Wang, Kun He, CuiYan Lin, Xian Cao, Jichuang Lai, Baohui Zeng, Xinmin Guo

**Affiliations:** a Department of Ultrasound, Guangzhou Red Cross Hospital of Jinan University, 510220 Guangzhou, China; b Department of Stomatology, Guangzhou Red Cross Hospital of Jinan University, 510220 Guangzhou, China

**Keywords:** Non-alcoholic steatohepatitis, Ultrasonics, Nanobubbles, Reactive oxygen species, Resveratrol

## Abstract

Non-alcoholic steatohepatitis (NASH) pathogenesis is primarily driven by lipotoxicity-induced oxidative stress and inflammation, yet effective treatments remain challenging to identify. In this work, a novel therapeutic approach was introduced via a ultrasound (US) −driven, reactive oxygen species (ROS) −scavenging and liver-targeted nanobubbles system, termed Apt-DTP-NBs@RSV@OCA, which co-encapsulated resveratrol (RSV) and obeticholic acid (OCA). This system provides a safe and efficient platform for specifically delivering these agents to the liver in the context of the NASH therapy. The synthesized nanobubbles showed a spherical morphology with an average diameter of 165 ± 6.05 nm, whose encapsulation efficiencies of approximately 93 % for RSV and 90 % for OCA were achieved. These nanobubbles exhibited the enhanced targeting and accumulation within NASH affected cells and the excellent biocompatibility in cytotoxicity experiments. Subsequently, *in vitro* assessments using HepG2 cells, Apt-DTP-NBs@RSV@OCA improved lipid metabolism and reduced ROS levels. It was also showed *in vivo* experiments in mice that the hepatic targeting of Apt-DTP-NBs@RSV@OCA increased their effective concentration within the liver. In addition, the hepatic-targeting and ultrasound-driving Apt-DTP-NBs@RSV@OCA nanocarriers enhanced the cellular uptake of RSV and OCA in a NASH cell model and improved ROS-scavenging capabilities. Meanwhile, these nanocarriers modulated lipid metabolism (triglycerides, total cholesterol), inflammatory cytokine metabolism (IL-4, IL-10, IL-15, TNF-α) and oxidative stress levels (SOD, MDA). Furthermore, mechanistic studies revealed that Apt-DTP-NBs@RSV@OCA activated the FXR/SHP signaling pathway, enhanced FoxO1 activity, and alleviated lipid accumulation, inflammation, and oxidative stress. In summary, these findings suggest that Apt-DTP-NBs@RSV@OCA pave a promising way for the treatment of NASH.

## Highlights

• Apt-DTP-NBs@RSV@OCA combines US activation, ROS scavenging, liver targeting and dual-drug delivery for NASH therapy.
• Features DTP-liposome shell, C3F8 core and aptamer surface for stable, targeted NASH cell delivery.
• Activates FXR/SHP pathway, reduces ROS/lipids, and improves inflammation/oxidative stress in NASH.

## 1 Introduction

Non-alcoholic steatohepatitis (NASH), characterised by excessive lipid accumulation and hepatocyte injury, is a common liver disease that poses significant therapeutic challenges due to its complex pathogenesis and the limited efficacy of single drug treatments [1]. Lipotoxicity has been identified as a central driver of NASH progression, primarily through its role in inducing oxidative stress and inflammation [2]. These processes contribute to hepatocyte injury and fibrosis, creating an urgent need for effective therapeutic strategies. Previous research has demonstrated that antioxidants possess the potential to ameliorate the pathology of NASH. This is achieved by reducing the generation of reactive oxygen species (ROS), modulating lipid metabolism, and alleviating inflammatory responses [3]. However, even though several antioxidants have advanced to clinical trial stages, they generally lack targeting specificity in application, resulting in limited effective drug uptake by liver tissues [4].

Resveratrol (RSV), a natural plant sirtuin 1 (SIRT1) activator, upregulates critical regulators of inflammatory and metabolic pathways in hepatocytes, such as the farnesoid X receptor (FXR). RSV has been shown to reduce ROS levels, suppress the synthesis and release of inflammatory cytokines, and alleviate oxidative stress-induced hepatocyte damage [5]. It has also shown significant protective effects in NASH mouse models through mechanisms including ROS inhibition, lipid regulation and anti-inflammatory activity [6,7]. Similarly, obeticholic acid (OCA), a highly selective FXR agonist, shows therapeutic potential for NASH by alleviating oxidative stress, reducing inflammation and preventing apoptosis. Clinical studies have confirmed its efficacy in ameliorating hepatic steatosis and fibrosis, highlighting its role in inhibiting NASH progression [8,9]. Mechanistically, OCA activates FXR and induces the expression of the small heterodimer partner (SHP), which suppresses key enzymes involved in lipid synthesis, reduces lipid deposition in the liver and enhances lipid transport. Together, these effects attenuate oxidative stress, reduce inflammation and improve fibrosis in NASH [[10], [11], [12]]. Additionally, FXR activation enhances FoxO1 transcriptional activity, which synergistically improves hepatic metabolism and immune responses [13,14]. However, during hepatocyte injury, FXR undergoes acetylation and subsequent cytoplasmic degradation, impairing the ability of OCA to effectively activate FXR and limiting its clinical efficacy. RSV, by activating SIRT1, reduces FXR acetylation, decreasing its cytoplasmic retention and degradation, thereby enhancing its transcriptional activity [15]. Therefore, combining RSV and OCA to synergistically target oxidative stress is a promising strategy to improve NASH outcomes, but the therapeutic potential of RSV and OCA is limited by their low bioavailability and inability to selectively target diseased liver tissue [16,17].

To overcome these limitations, this study proposes the development of ultrasound (US) −driven nanobubble drug delivery system functionalised with a NASH-specific aptamer. This system enables precise, controlled drug release through the synergistic interaction of aptamer targeting and US-triggered mechanisms. Furthermore, in this study, DSPE-TK-PEG (DTP) was incorporated into the synthesized nanobubbles. Due to the presence of a thioketal (TK) bond in its structure, it was rendered highly sensitive to ROS. Upon reaching areas of high ROS concentration, such as inflammatory regions, the TK bond in the DTP moiety rapidly react with ROS to trigger drug release. This mechanism not only enhances the precision of drug delivery, ensuring effective treatment of the diseased site, but also minimizes damage to healthy tissues [18].

**Scheme 1:**
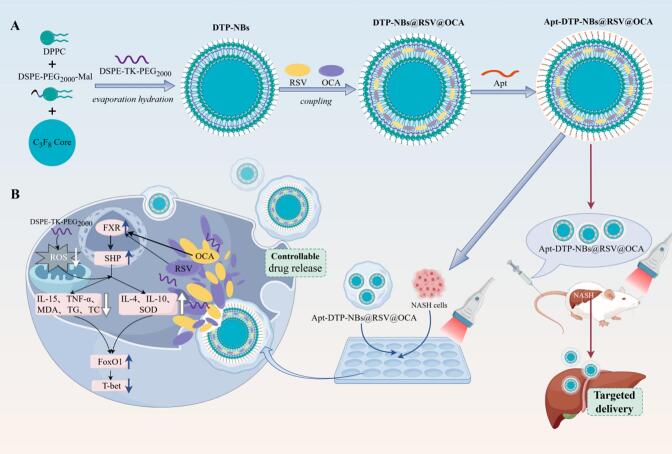
Schematic representation of the preparation process for Apt-DTP-NBs@RSV@OCA and its mechanism in NASH-targeted therapy.

In this study, the nanobubble consists of a DTP-modified liposome shell encapsulating a perfluoropropane (C_3_F_8_) core, with the surface of the nanobubble functionalized with Aptamers (Apt) to achieve targeting of NASH cells. Under the action of Apt, the prepared nanobubbles can effectively enter NASH cells and upon ultrasound mediation, release an ultrasound contrast agent (C_3_F_8_), therapeutic agents (RSV and OCA), and a reactive oxygen species scavenger (DTP). *In vitro* evaluations using HepG2 cells demonstrated that Apt-DTP-NBs@RSV@OCA improved lipid metabolism, and reduced ROS levels. The application of ultrasound energy results in the evaporation of C_3_F_8_, causing the nanobubbles to undergo expansive collapse, subsequently disrupting their structure. This disruption facilitates the release of RSV and OCA carried by the nanobubbles.thereby enhancing the therapeutic efficacy against NASH. This study demonstrates that synthetic nanobubbles can enhance the regulation of the following aspects: lipid metabolism (including triglycerides and total cholesterol), inflammatory cytokine metabolism (involving IL-4, IL-10, IL-15 and TNF-α), and oxidative stress levels (specifically SOD and MDA). In a NASH cellular model, Apt-DTP-NBs@RSV@OCA nanocarriers enhanced cellular uptake of RSV and OCA, and improved ROS scavenging capacity. Additionally, in this study, it was demonstrated that the FXR/SHP signaling pathway was activated by Apt-DTP-NBs@RSV@OCA, the activity of FoxO1 was enhanced (Scheme 1). Based on the above research, the aim was to construct an Apt-modified, ROS-scavenging, and US-driven nanobubble system capable of co-encapsulating RSV and OCA.This nanocarrier provides a stable platform for the targeted delivery of antioxidant drugs to NASH cells, offering a novel approach to improve therapeutic outcomes in NASH.

## 2 Materials and methods

### 2.1 Materials

1,2-Distearoyl-*sn*-glycero-3-phosphoethanolamine-tk- (polyethyleneglycol) (DSPE-TK-PEG, DTP), 1,2-distearoyl-*sn*-glycero-3-phosphoethanolamine-N-[succinimidyl (polyethyleneglycol) − 2000] (DSPE-PEG-2K-NHS), Stone Plastic Composites (SPC), Cholesterol, Resveratrol (RSV), Obeticholic Acid (OCA) and perfluoropropane gas (C_3_F_8_) were purchased from Xi’an ruixi Biological Technology Co., Ltd (Xi’an, China). Polycarbonate Film was purchased from Whatman. Dulbecco’s Modified Eagle Medium and Fetal Bovine Serum (FBS) was obtained from Gibco. The NASH aptamer sequence labeled with FITC was purchased from Synbio Technologies (Shanghai, China). Unless otherwise specified, all reagents and solvents used were of analytical grade and did not require any additional purification. HepG2 cells (human HCC cell line) were purchased from the Shanghai Cell Bank of the Chinese Academy of Sciences. The cells were cultured in Dulbecco's Modified Eagle Medium (DMEM), supplemented with 10 % fetal bovine serum (FBS) and 100 units per milliliter of penicillin–streptomycin, within a humidified incubator set at 37 degrees Celsius and containing 5 % carbon dioxide.

### 2.2 Preparation of NBs

#### 2.2.1 Synthesis of ultrasonic nanobubbles

SPC, DSPE-TK-PEG (DTP) and cholesterol were dissolved together in (3 mL), then transferred to a round-bottom flask and evaporated under reduced pressure to form a film. The film was hydrated with deionized water and, under water bath ultrasonication, perfluoropropane gas was introduced into the solution for 10 min. The solution was then treated with a liposome extruder (using a 200 nm filter membrane). Finally, additional deionized water was added to adjust the volume to 10 mL, thereby obtaining the DTP@NBs solution.

#### 2.2.2 Aptamer-functionalized ultrasonic nanobubbles

DSPE-PEG-2K-NHS was reacted with the NASH aptamer (Apt) at room temperature for four hours, and then purified by dialysis to remove the uncoupled DSPE-PEG-2K-NHS, thereby obtaining the DSPE-PEG-2K-Apt. SPC, DTP-2K, cholesterol, RSV, and OCA were dissolved together in chloroform (3 mL), then transferred to an eggplant-shaped flask and evaporated under reduced pressure to form a film. DSPE-PEG-2K-Apt was added to hydrate the film, and C_3_F_8_ was bubbled into the solution under water bath ultrasound conditions for 10 min. The mixture was then processed through a liposome extruder (with a 200 nm filter membrane). Finally, deionized water was added to make the total volume up to 10 mL, thereby obtaining the Apt-DTP-NBs@RSV@OCA.

### 2.3 Characterizations of NBs

The morphological characteristics of the prepared Apt-DTP-NBs@RSV@OCA were investigated using a transmission electron microscope (TEM). Facilitate TEM imaging, Apt-DTP-NBs@RSV@OCA were first dispersed in deionized water at a concentration of 5 mg/mL. Subsequently, 5 mL of this prepared solution was carefully transferred onto a perforated carbon-coated copper TEM grid. Following air drying, a state-of-the-art high-resolution TEM instrument (H-800, Hitachi, Japan) was employed to capture detailed images of the samples, operating at an accelerating voltage of 200 kV. The composition of elemental constituents within the formulated Apt-DTP-NBs@RSV@OCA was examined utilizing the Energy Dispersive System (EDS). The size, zeta potential, and polydispersity index (PDI) of the LNPs were determined using dynamic light scattering with a NanoBrook 90plus PALS from Brookhaven Instruments (US). For a comprehensive understanding of the nanoparticles’ characteristics, the Structures and properties were meticulously determined utilizing a UV Absorption Spectroscopy and fluorescence spectrometer manufactured by Beijing Pulse Instruments.

### 2.4 RSV and OCA encapsulation efficiency (EE) of drug-loaded NBs

The content of RSV and OCA loaded was determined by ultraviolet (UV) measurements. A freshly prepared solution of RSV and OCA was used to construct standard curve. Samples with 0.5, 1, 1.5 and 2 mg of RSV or OCA, respectively, were added to the lipid mixture to produce drug-loaded NBs. After shaking the NB suspensions were centrifuged at 800 rpm for 3 min. Next, solutions containing free RSV and OCA were collected, and RSV and OCA concentrations were determined on a UV–visible spectrometer (The excitation wavelength is 450 nm; the emission wavelength is 521 nm.). The amounts of uncombined apatinib were obtained by absorption measurements using a standard curve. EE (%) was calculated using the following formula:

$$Drug\;EE\;=\frac{Total\;RSV\;(OCA)-\;free\;RSV\;(OCA)}{Total\;RSV\;(OCA)}\times 100\%$$

### 2.5 Establishment of a cell model of non-alcoholic steatohepatitis

HepG2 cells were cultured with free fatty acid (FFA) DMEM for approximately 48 h. FFA components were composed of palmitic acid and oleic acid (Sigma-Aldrich) at a ratio of 1:2 and diluted with FBS-free DMEM to a final concentration of 4 mM. The cells were stained with oil red O to confirm fat deposition.

### 2.6 Cell viability and apoptosis assays

#### 2.6.1 CCK8 analysis of the of Apt-DTP-NBs@RSV@OCA

Prepared cell suspensions were seeded into 96-well plates and incubated for 24 h. Subsequently, the cells were treated with Apt-DTP-NBs@RSV@OCA at concentrations of 0, 9.09, 18.18, 27.27, 36.36 and 45.45 μg/mL, according to experimental groups, for 24 and 48 h. Ten microliters of CCK8 solution were added to each well, and the plates were incubated within the incubator for an additional 2 h. The absorbance of each sample well at 450 nm was measured using a microplate reader (Molecular Devices, USA). The experiment was repeated three times.

#### 2.6.2 US-driven ROS-scavenging nanobubbles loaded with RSV and OCA for the treatment of NASH

The cytotoxicity of the formulated NBs on hepatic cell line (HepG2 cells and NASH cells) was assessed using the CCK-8 assay. The cells were incubated in 96-well plates at a density of 1 × 104 cells per well at 37℃. After 24 h, cells were treated with different concentrations of NBs solutions at 37 °C. The specific experimental groups were as follows: (a) HepG2 cells (Control group); (b) NASH cells; (c)NASH cells + 10 μg/mL NBs@RSV; (d) NASH cells + 20 μg/mL NBs@RSV; (e) NASH cells + 9.09 μg/mL NBs@OCA; (f) NASH cells + 18.18 μg/mL NBs@OCA; (g) NASH cells + 8.36 μg/mL DTP-NBs@RSV@OCA；(h) NASH cells + 16.72 μg/mL DTP-NBs@RSV@OCA；(i) NASH cells + 9.09 μg/mL Apt-DTP-NBs@RSV@OCA; (j) NASH cells + 18.18 μg/mL Apt-DTP-NBs@RSV@OCA; (k) NASH cells + 9.09 μg/mL DTP@NBs;(l) NASH cells + 18.18 μg/mL DTP@NBs. All groups were performed by US irradiation (SP100 sonoporator, Sonidel Ltd, USA). The optimal intensity of ultrasound operating parameters was set at a power level of 1.6 W/cm^2^, a frequency of 1 MHz, and a 50 % duty cycle for 60 s. After 24 h or 48 h incubation, CCK-8 solution was then added to each well, and the cells were incubated for an additional 2 h at 37℃. The absorbance of each sample well at 450 nm was measured using a microplate reader (Molecular Devices, USA). The experiment was repeated three times.

### 2.7 Binding affinity of Apt-DTP@NBs@RSV@OCA for NASH cells *in vitro*

#### 2.7.1 Measurement of lipid levels

The specific experimental groups were as follows: (a) HepG2 cells (Control group); (b) NASH cells; (c) NASH cells + DTP-NBs; (d) NASH cells + DTP-NBs@RSV@OCA; (e) NASH cells + Apt-DTP-NBs@RSV@OCA. First, the lipid deposition in cells of each group was observed using Oil Red O staining. Then, the cells in the group were seeded onto a 6-well plate and cultured for 24 h. All groups were performed by US irradiation. Following this, the cells underwent fixation, washing, isopropanol rinsing, staining, and nuclear counterstaining. Lipid droplet formation was observed under a microscope, with photographs taken at 200x and 400x magnifications. The area or number of lipid droplets was measured to assess the impact of the nanocarriers on lipid droplet formation.

#### 2.7.2 Cellular uptake of nanoparticles *in vitro*

Cells were initially distributed into multi-well plates with a capacity of twelve wells each, containing 1 × 105 cells per well. After an incubation period of 24 h, a combination of DTP-NBs/ DTP-NBs@RSV@OCA/ Apt-DTP-NBs@RSV@OCA was introduced into the wells. All groups were performed by US irradiation. These nanoscale particles were cultivated and treated with a FITC probe for visualization, while the cell nuclei were counterstained using Mayer's Hematoxylin Stain Solution. Following 2 h incubation period, the cells underwent triple washing with PBS to eliminate any residual free FITC-labeled nanoparticles. Photomicrographs were captured employing fluorescence imaging methodologies.

### 2.8 Verification of the targeting ability of nanoparticles *in vivo*

The murine NASH model was established through the administration of a Methionine/ choline −deficient (MCD) diet to 8 weeks old mice for a period of 4 weeks, following established protocols. Mice were randomly divided into 2 groups (n = 3 per group). The fluorescent probe FITC −conjugated drug was introduced into the mice intravenously through the tail vein. The two groups of drugs administered included Apt-DTP-NBs@RSV@OCA and DTP-NBs@RSV@OCA. The fluctuations in fluorescence intensity distribution *in vivo* were evaluated across different time intervals (0, 2, 6, and 24 h) utilizing the IVIS imaging system (AniView 100, BioLight, Guang zhou, China). For organ imaging, the euthanasia of mice was carried out, followed by the removal of liver, spleen, kidney, heart, lung organ tissues and the fluorescence were captured.

### 2.9 Cell apoptosis after US irradiation

Cellular apoptosis was evaluated through the application of FITC Annexin V and propidium iodide staining, subsequently analyzed using flow cytometry. The HepG2 cells were seeded into 24 −well culture plates at a density of 1 × 104 cells per well and allowed to incubate for 24 h. Subsequently, the cells were treated with nanomaterials following the group and US irradiation was performed. The specific experimental groups were as follows: (a) HepG2 cells (Control group); (b) NASH cells; (c) NASH cells + DTP@NBs; (d) NASH cells + NBs@RSV; (e) NASH cells + NBs@OCA; (f) NASH cells + DTP-NBs@RSV@OCA; (g) NASH cells + Apt-DTP-NBs@ RSV@OCA; The Specimens were obtained utilizing Novocyte 2040R equipment and subsequently examined Through the application of FlowJo software.

### 2.10 Detection of ROS in cells

Seed HepG2 cells in a 6-well plate one day in advance and incubate them for 24 h. Prepare FFA (with a palmitic acid to oleic acid ratio of 1:2 and a final concentration of 2 mM) to treat HepG2 cells for 48 h to establish a NASH model. Add corresponding drugs according to the grouping and continue incubating for 24 h. Subsequently, all groups were performed by US irradiation. Add a 1:1000 dilution of ROS probe and incubate in the dark for 20 min. Wash the cells three times with blank culture medium to remove excess probe. If applicable, add a positive probe (at a ratio of 2:1000), incubate for 30 min, and then wash three times again. Digest the cells, wash them twice with PBS, centrifuge to discard the supernatant, and resuspend the cells in 200 μl of PBS. Analyze the intracellular ROS levels using a flow cytometer near the excitation wavelength of 535 nm and emission wavelength of 610 nm. Use Novocyte 2040R software to analyze the results.

### 2.11 *In vitro*, Apt-DTP-NBs@RSV@OCA can improve lipid metabolism, inflammatory cytokines metabolism and oxidative stress levels in NASH

#### 2.11.1. Enzyme-linked immunosorbent assay

The triglyceride levels, hepatic function and inflammatory factor were quantified using enzyme-linked immunosorbent assay (ELISA) employing a double antibody one step sandwich method. The ELISA test kit involved the sequential addition of specimens, standard products, and HRP-labeled detection antibodies, followed by incubation and Thoroughwashing steps. Absorbance (OD values) at a wavelength of 450 nm was measured using an enzyme marker. Calibration standards were run simultaneously with samples to generate a standard curve correlating Optical Density with activity. The activity of the samples was subsequently determined by comparing their OD values to the standard curve.

#### 2.11.2 Real-time polymerase chain reaction (RT-PCR)

RT-PCR was conducted using a CFX Real-Time PCR System (Bio-Rad, Hercules, CA, USA). Extraction of total RNA from cells was done with the use of a TRIzol Reagent Kit (Invitrogen, Life Technologies, Rockville, MD). cDNA was prepared using a commercially available kit (Thermo Fisher Scientific,Waltham, USA). RT-PCR was performed using SYBR Green PCR Master Mix (Thermo Fisher, Waltham, USA) with gene specific primers, and the relative gene expression profiles were analyzed by the ΔΔCt method using 18S rRNA. GAPDH served as a normalization gene. The primers list is provided in Supplementary Table S1. Each PCR reaction was performed in triplicate, and the comparative analysis was performed using the 2^−△△Ct^ method.

#### 2.11.3 Western blotting experiment

Protein extraction from cellular sources was conducted, with subsequent determination of protein concentrations facilitated by employing the BCA Enhanced Protein Assay Kit (BCA, Beyotime, Shanghai, China). Following extraction, samples underwent heat treatment and were subsequently loaded onto SDS-PAGE for electrophoresis. Post Electrophoresis, the samples were transferred onto a nitrocellulose membrane using a transfer buffer. Following this, the membrane was incubated at room temperature for 1 h with primary antibodies against SIRT1, FXR, SHP, FoxO1 and T-bet. Subsequently, the Nitrocellulose membrane underwent blocking with 5 % non-fat milk (w/v) dissolved in phosphate-buffered saline with Tween 20 and was then subjected to incubation with specific primary antibodies for a duration of 4–6 h, followed by secondary antibodies for 2 h at ambient temperature. The acquisition of images and subsequent quantitative analysis were carried out utilizing the Odyssey imaging system (Li-Cor, NE, USA).

### 2.12 Statistical analysis

Statistical analyses were performed using one-way analysis of variance (ANOVA) (SPSS Software, version 20.0; IBM Inc, Armonk, NY, USA). All data are reported as the mean ± SD. The statistical significance level was set at 0.05.

## 3 Results and discussion

The development of a safe and effective drug delivery system for the precise targeting and release of resveratrol (RSV) and obeticholic acid (OCA) to non-alcoholic steatohepatitis (NASH) lesion sites is considered crucial, as it is expected to enhance drug targeting, reduce systemic toxicity, and improve clinical outcomes. Given the complexity of NASH, limited benefits are offered by monotherapy. Through combination therapy with antioxidant drugs, key regulators of hepatocyte inflammation and injury can be activated, hepatocyte function can be improved, and inflammation can be suppressed, thereby maximizing therapeutic effects and achieving clinical endpoints for NASH improvement [19,20]. In this study, we developed Apt-targeted, US-driven and ROS-scavenging nanobubbles (NBs) system, designated as Apt-DTP-NBs@RSV@OCA, specifically for the targeted delivery and controlled release of RSV and OCA, aiming to overcome the challenges associated with their low bioavailability and difficulty in specifically targeting NASH lesion sites.

### 3.1 Design and characterization of liver −targeted Apt-DTP-NBs@RSV@OCA nanocarriers

**Fig. 1:**
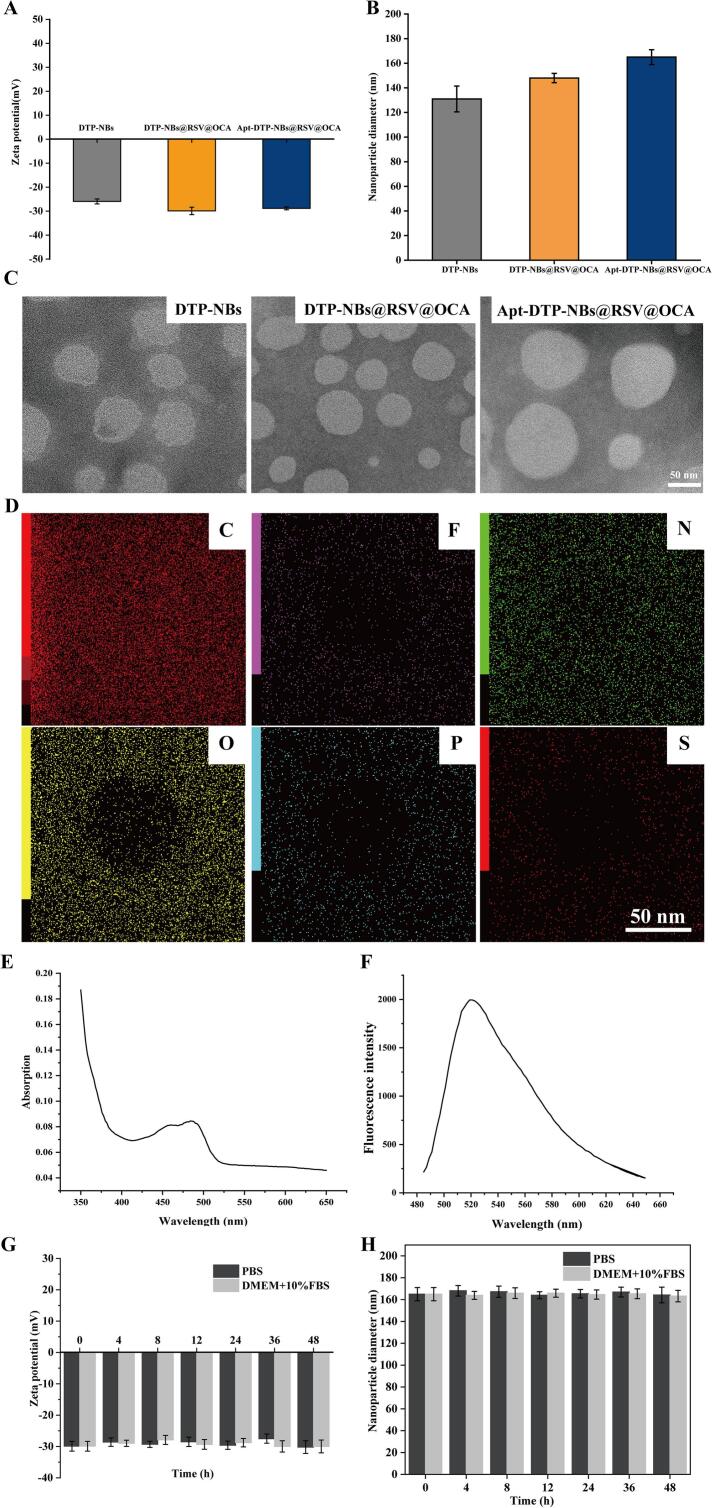
Characterization of prepared nanobubbles. (A) Zeta potential, (B) Size distribution and (C) TEM images of DTP@NBs, DTP-NBs@RSV@OCA, and Apt-DTP-NBs@RSV@OCA. (D) EDS analysis of Apt-DTP-NBs@RSV@OCA, (E) UV–Vis spectra and (F) Fluorescence spectra of Apt-DTP-NBs@RSV@OCA. (G) Zeta potential and (H) Size distribution of Apt-DTP-NBs@ RSV@OCA after 48 h.

As shown in (Fig. 1A), the size distribution and zeta potential of the DTP −NBs, DTP −NBs@RSV@OCA and Apt-DTP-NBs@RSV@OCA were measured. The zeta potentials were −25.94 ± 1.04 and −29.91 ± 1.56 mV in DTP-NBs and DTP-NBs@RSV@OCA, respectively, while the zeta potential in Apt-DTP-NBs@RSV@OCA was −28.84 ± 0.61 mV. Changes in zeta potential indicated the successful preparation of NBs loaded with Aptamer, OCA, and RSV. The average particle sizes and PDI of DTP@NBs, DTP-NBs@RSV@OCA, and Apt-DTP-NBs@RSV@OCA were 131 ± 10.5 nm (PDI (Polydispersity Index): 0.17 ± 0.015), 148 ± 3.79 nm (PDI: 0.18 ± 0.02), and 165 ± 6.05 nm (PDI: 0.17 ± 0.01), respectively, as shown in (Fig. 1B). TEM observations showed that DTP@NBs, DTP@NBs@RSV@OCA and Apt-DTP@RSV@OCA@NBs were spherical with similar morphological (Fig. 1C). The EDS image analysis of Apt-DTP-NBs@RSV@OCA reveals that it contains the element C, O, P, S, F, N (Fig. 1D), and its encapsulation efficiencies for RSV and OCA reach 91 % (Fig. 1E) and 92 % (Fig. 1F), respectively. Our results demonstrate that the Apt-DTP-NBs@RSV@OCA exhibit good dispersion stability and a suitable size for effective delivery, as characterized by TEM, nanoparticle size and zeta potential analysis. In this study, the developed nanobubbles exhibited excellent stability in terms of particle size and zeta potential for at least 48 h when dispersed in PBS solution containing 10 % FBS at 37 °C (Fig. 1G and H). This finding further supports the feasibility of their application in the treatment of NASH.

### 3.2 Cell viability and apoptosis assays

#### 3.2.1 CCK8 analysis of the of Apt-DTP-NBs@RSV@OCA

**Fig. 2:**
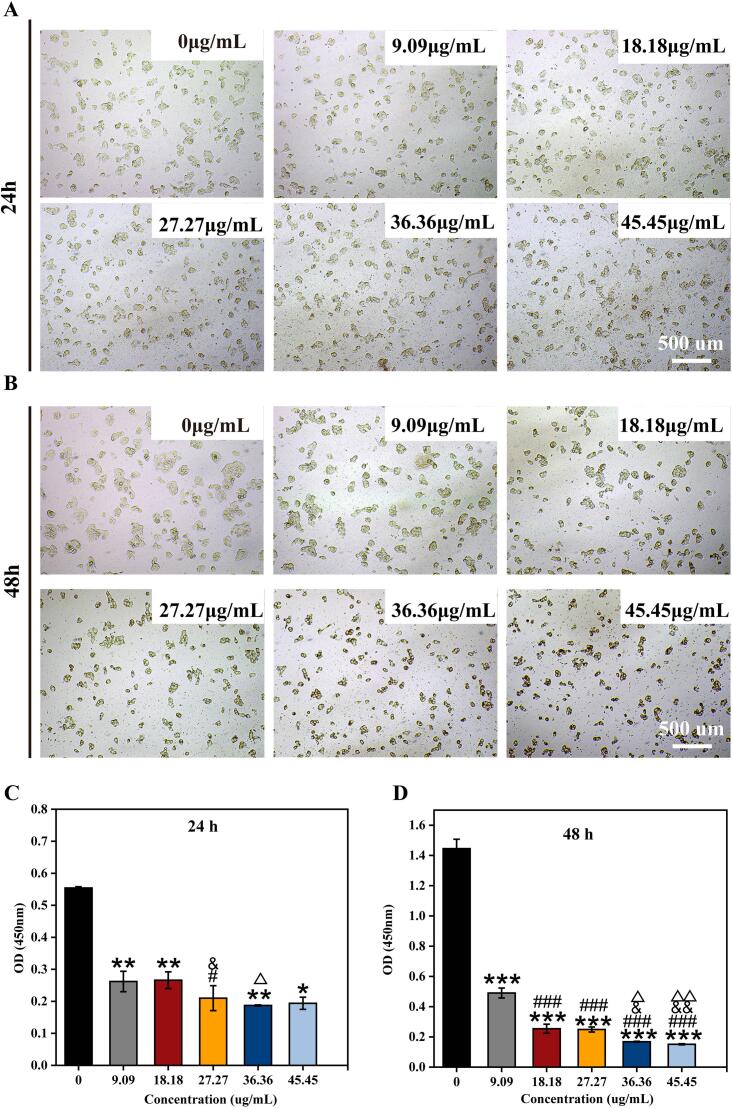
Effects of varying concentrations (0–45.45 μg/mL) on HepG2 cells after 24 h (A) and 48 h (B) (CCK-8 assay). OD value statistical chart at (C) 24 h and (D) 48 h. Values shown are means ± SD, ***p < 0.001 vs. Control, **p < 0.01 vs. Control, ###p < 0.001 vs. 9.09, #p < 0.05 vs. 9.09, ##p < 0.01 vs. 9.09, &p < 0.05 vs. 18.18, &&p < 0.01 vs. 18.18, △p < 0.05 vs. 27.27, △△p < 0.05 vs. 27.27, n = 3.

Synthetic nanobubbles interact with targeted tissues during their application. An ideal nanobubble should exhibit low levels of cytotoxicity to ensure safety [21]. Initially, the cell viability was analyzed at various concentrations of Apt-DTP@NBs@RSV@OCA (0, 9.09, 18.18, 27.27, 36.36, 45.45 μg/mL) when cocultured with HepG2 cells (Fig. 2A). The results indicated that, after 24 h of coculture, compared to the control, the concentrations of 9.09 μg/mL and 18.18 μg/mL had minimal effects on cell viability, whereas at concentrations ≥ 27.27 μg/mL, there was a decline in cell viability (Fig. 2B). After 48 h of coculture with HepG2 cells, the cell viability in the 9.09 μg/mL group increased compared to the previous level, while there were no significant changes in cell viability observed in the other groups. It is demonstrated that at a concentration of 9.09 μg/mL, Apt-DTP-NBs@RSV@OCA still exhibits a proliferative effect on HepG2 cells (Fig. 2C). Therefore, without compromising cell viability and drug concentration efficacy, concentrations of 9.09 μg/mL were selected for subsequent cellular therapy studies in NASH.

#### 3.2.2 US-driven ROS-scavenging nanobubbles loaded with RSV and OCA for the treatment of NASH

**Fig. 3:**
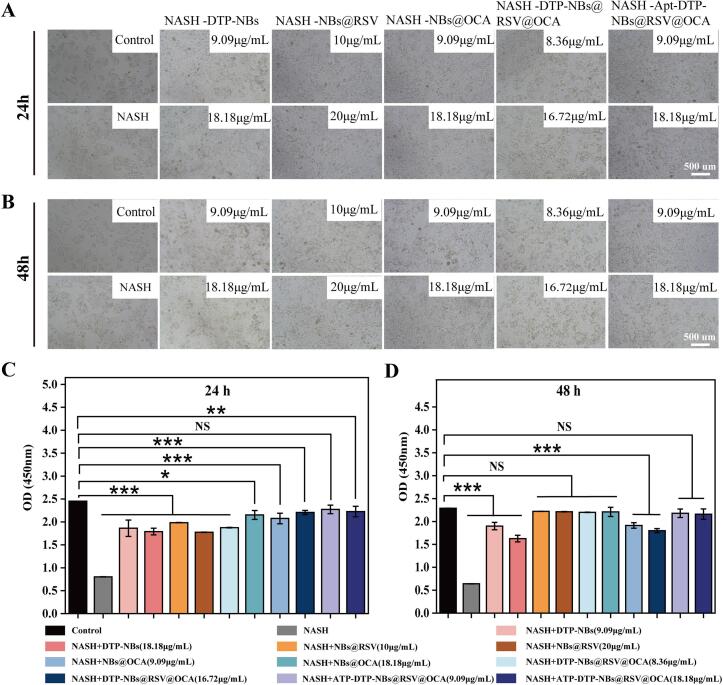
Cytocompatibility of nanobubbles *in vitro*. (A) Effects of varying nanomaterial concentrations on HepG2 cells at 24 h. (B) Effects of varying nanomaterial concentrations on HepG2 cells at 48 h. (C) OD value statistical chart at 24 h. (D) OD value statistical chart at 48 h. Values shown are means ± SD, *p < 0.05, **p < 0.01, ***p < 0.001 indicate statistical significance, n = 3.

As shown in (Fig. 3A) and (Fig. 3B), these cells were cocultured with various nanocarriers at different concentrations, and cell viability was assessed following the co-incubation period. The experimental results indicated that after 24 h of co-culture (Fig. 3C), no significant difference in cell proliferation rate was observed between the targeted nanomedicine at a concentration of 9.09 μg/mL and the normal group. In contrast, NBs@RSV and NBs@OCA, at the same concentrations (9.09 μg/mL and 18.18 μg/mL), required 48 h of co-culture (Fig. 3D) to achieve a proliferation rate comparable to that of the normal group. This demonstrated that the targeted nanomedicine Apt-DTP@NBs@RSV@OCA was able to accelerate drug absorption, thereby exerting its effects more rapidly. In addition, after 48 h of co-culture (Fig. 3D), no significant difference in proliferation rate was still observed between the targeted nanomedicine at a concentration of 9.09 μg/mL and the normal group. This result further indicated that Apt-DTP@NBs@RSV@OCA not only accelerated drug absorption but also restored the cell state to a level comparable to that of the normal group.

### 3.3 Binding affinity of Apt-DTP@NBs@RSV@OCA for NASH cells *in vitro*

#### 3.3.1 Measurement of lipid levels

**Fig. 4:**
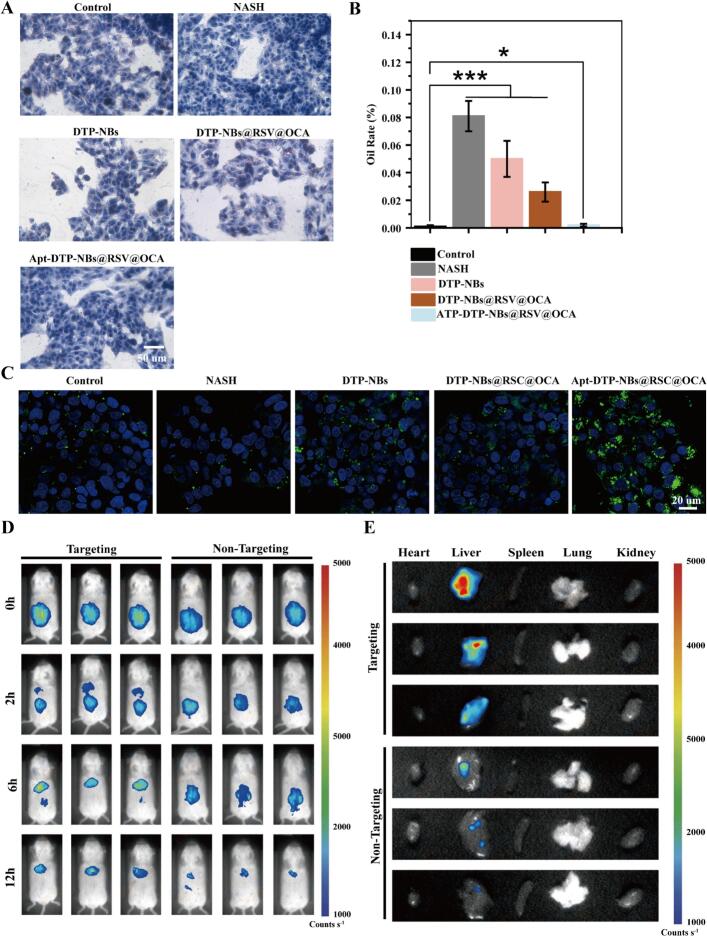
(A) Oil Red O staining of nanoparticles. Scale bar = 50 µm (B) Oil Rate (%) Statistical Chart (C) FITC-labeled nanoparticles (green) with nuclei stained in blue (Mayer's hematoxylin). Scale bar = 20 µm. (D) IVIS images showing distribution of Apt-DTP-NBs@RSV@OCA (Targets A-C) and DTP-NBs@RSV@OCA (Targets D-F) at 0, 2, 6, and 24 h post-injection *in vivo*. (E) Ex vivo fluorescence images of heart, liver, spleen, lungs, and kidneys to observe organ-specific signal distribution. (For interpretation of the references to colour in this figure legend, the reader is referred to the web version of this article.)

To explore the Binding affinity of Apt-DTP-NBs@RSV@OCA for NASH cells *in vitro*, HepG2 cells were exposed to a free fatty acid (FFA) mixture of oleate and palmitate at ratio of 2:1 to induce cellular steatosis. The deposition of lipids among cells can be observed through the Oil Red O staining experiment (Fig. 4A). As expected, Oil Red O staining and quantitative colorimetric assays showed that the lipid accumulation was significantly decreased in steatotic HepG2 cells treated with Apt-DTP-NBs@RSV@OCA than control groups for 24 h (Fig. 4B).

#### 3.3.2 Cellular uptake of nanoparticles *in vitro*

Under microscopic observation, the formation of lipid droplets was shown to be minimal in the presence of Apt-DTP-NBs@RSV@OCA. Specifically, the oil red ratio (%) was measured to be 0.002 ± 0.001 at 24 h. Furthermore, the reduction in lipid accumulation by Apt-DTP-NBs@RSV@OCA was confirmed by a decrease in TG levels in HepG2 cells. In our study, fluorescence microscopy (Fig. 4C) revealed that the fluorescent signal from the targeted nanocarrier was primarily localized around NASH cells, compared to the control group. This finding indicates that Apt-DTP-NBs@RSV@OCA exhibits effective targeting ability against NASH *in vitro*.

### 3.4 Verification of the targeting ability of nanoparticles *in vivo*

We investigated the biodistribution (specifically liver-targeting ability) of Apt-DTP-NBs@RSV@OCA nanocarriers using a mouse model. To accomplish this, both free Apt-DTP-NBs@RSV@OCA and DTP-NBs@RSV@OCA were administered to mice via tail vein injection. Subsequently, the distribution of FITC fluorescence was tracked using an *in vivo* imaging system at various time points (0, 2, 6, and 12 h) (Fig. 4D). It is anticipated that all these nanocarriers will be rapidly absorbed by hepatic tissues, with their fluorescence intensity reaching a peak shortly thereafter.

Notably, Apt-DTP-NBs@RSV@OCA exhibited the stronger fluorescence intensity in formulations. Specifically, the intensity of Apt-DTP-NBs@RSV@OCA was greater than that of DTP-NBs@RSV@OCA at 0 h and gradually declined over 12 h. This indicated that Apt-DTP-NBs@RSV@OCA demonstrated the highest specificity for the liver and an extended retention time within it. Additionally, Apt-DTP-NBs@RSV@OCA could be metabolized and cleared from the liver. The extended blood circulation of Apt-DTP-NBs@RSV@OCA facilitated the transportation and accumulation of RSV and OCA in the liver. Furthermore, the conjugation of aptamers to DTP contributed to their guidance towards the liver. These findings demonstrated that Apt-DTP-NBs@RSV@OCA nanocarriers could effectively accumulate in the liver and exhibited excellent biocompatibility *in vivo*.

A few hours after injection, the mice were euthanized. Subsequently, tissues from the heart, liver, spleen, lungs, and kidneys were collected from each group of mice. Then, these exvivo organs were imaged using the IVIS imaging system to observe the specific distribution of fluorescent signals in each organ. Major organs including the liver, heart, spleen, lung and kidney were collected for fluorescence imaging at (0, 2, 6 and 12 h) after injection. FITC-labeled nanoparticles were found to mainly accumulate in the liver (Fig. 4E). The nanoparticle also deposited in the spleen and lung to some extent. The fluorescence intensity was highest in the liver at all the time point. These results indicate that the nanoparticles synthesized in this study, loaded with drugs, primarily accumulate in the liver.

### 3.5 Cell apoptosis after US irradiation

**Fig. 5:**
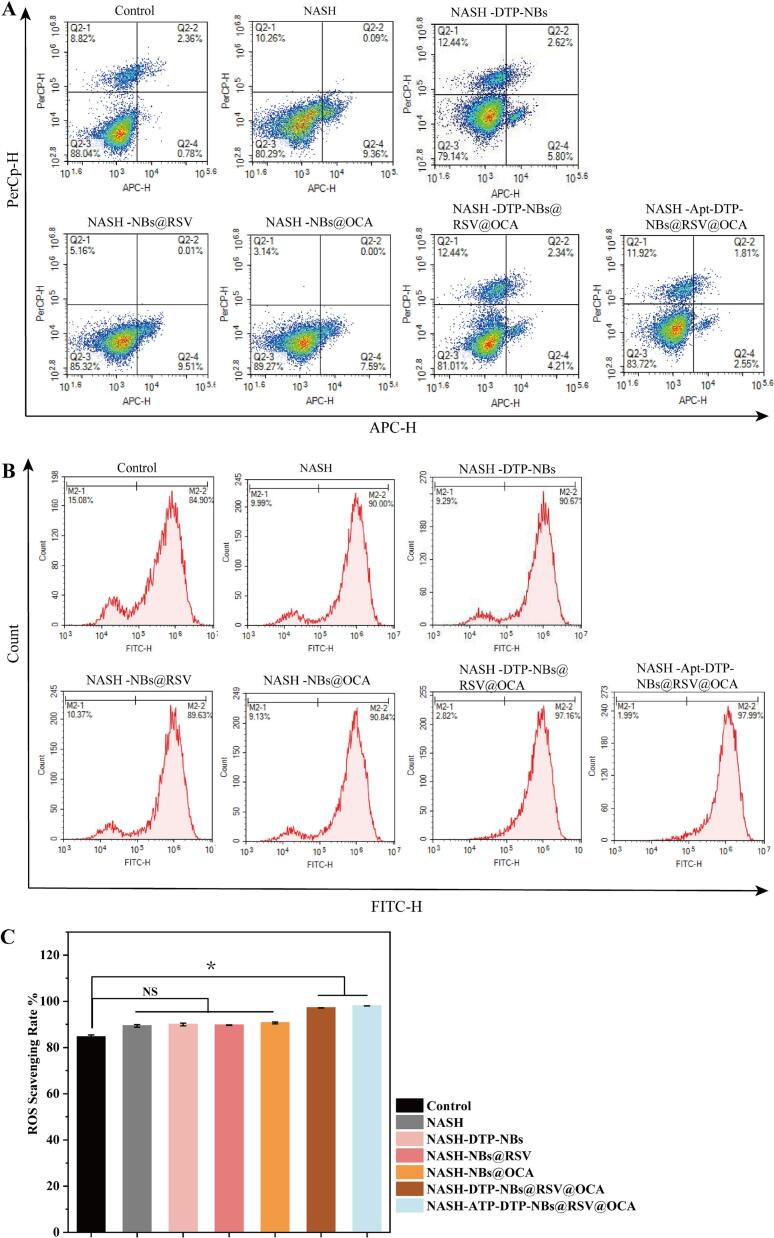
(A) Effect of Apt-DTP-NBs@RSV@OCA on apoptosis in NASH cells. Flow cytometry analysis 24 h post-treatment. (B) Effect of Apt-DTP-NBs@RSV@OCA on ROS levels in NASH cells. Flow cytometry results showing intracellular ROS levels post-treatment. (C) ROS Scavenging Rate (%) Statistical Chart.

Previous results have demonstrated that Apt-DTP-NBs@RSV@OCA possesses excellent targeting ability towards hepatocytes. Apoptotic rates in different treatment groups were determined by flow cytometry (Fig. 5A). Accordingly, the apoptotic rate in the Apt-DTP-NBs@RSV@OCA + US treatment group was 4.66 %±0.3 %. The experimental results indicate that no significant difference in cell apoptosis rate is observed between the Apt-DTP-NBs@RSV@OCA + US group and the control group, demonstrating that the nanobubbles are characterized by good biocompatibility and safety as drug delivery carriers.

### 3.6 Detection of ROS in cells

During the progression of NASH, high blood sugar levels and the accumulation of lipids in the liver stimulate the overproduction of ROS in hepatocytes [22,23]. This leads to an imbalance of ROS and results in mitochondrial dysfunction. Studies have shown that the hepatic FXR-SHP signaling pathway is associated with insulin resistance, hepatic lipid accumulation, and fibrosis caused by NASH [[24], [25], [26], [27]]. Activation of this signaling pathway primarily alleviates NASH by reducing hepatic de novo lipogenesis and collagen deposition. However, it's important to note that solely activating the FXR-SHP signaling pathway may have limitations in treating NASH, as it does not effectively mitigate oxidative stress and inflammation in the liver, which are crucial factors in the pathogenesis of NASH. Oxidative stress initiates inflammation and plays a pivotal role in the development of NASH [28].

Research has demonstrated that RSV, an effective agonist of SIRT1 derived from natural plants, can upregulate the FXR, a key regulator in the metabolic pathways of hepatocyte inflammation and injury, thereby reducing ROS, inhibiting the synthesis and release of inflammatory cytokines, and alleviating oxidative stress and hepatocyte damage [29]. Previous studies have confirmed that the combination of RSV and ROS scavengers can synergistically treat non-alcoholic fatty liver disease [30]. On the other hand, OCA as a highly selective agonist of FXR, can inhibit oxidative stress, decrease inflammation and apoptosis levels by activating FXR. Furthermore, clinical trials have further validated its efficacy in improving steatosis and liver fibrosis in NASH, thereby inhibiting the progression of NASH [5,31]. However, their clinical application is hindered by its physical and chemical characteristics.

To validate the effectiveness of Apt-DTP-NBs@RSV@OCA in reducing ROS levels in NASH cells, flow cytometry analysis was conducted in this study. The experimental setup included a control group (HepG2 cells with US stimulation, HepG2 + US), a disease control group (NASH cells with ultrasound stimulation, NASH + US), and five treatment groups utilizing different sizes and compositions of nanoparticles combined with US stimulation.

Flow cytometry results indicated that, compared to the disease control group (Fig. 5B), ROS −scavenging capability was observed in all treatment groups. Notably, the strongest ROS −scavenging capability was demonstrated in the Apt-DTP-NBs@RSV@OCA combined with ultrasound stimulation group. Specifically, the ROS scavenging capability of this group was found to be superior to that of all other treatment groups, with the difference being statistically significant (*P* < 0.05) (Fig. 5C). Furthermore, when NBs@RSV and NBs@OCA were used individually with ultrasound stimulation, they also showed a certain degree of ROS scavenging capability, but their efficacy was not as pronounced as that of the Apt-DTP-NBs@RSV@OCA combination. Similarly, the DTP-NBs@RSV@OCA and DTP-NBs groups also exhibited ROS scavenging capability but failed to match the reduction achieved by the Apt-DTP-NBs@RSV@OCA group.

In summary, this study's flow cytometry analysis revealed that Apt-DTP-NBs@RSV@OCA reduces ROS levels in NASH cells, particularly when used at specific sizes and compositions combined with ultrasound stimulation. The potential application of Apt-DTP-NBs@RSV@OCA in the treatment of NASH is supported by this result.

### 3.7 *In vitro*, Apt-DTP-NBs@RSV@OCA can improve lipid metabolism, inflammatory cytokines metabolism and oxidative stress levels in NASH

#### 3.7.1 Enzyme-linked immunosorbent assay

Studies have shown that RSV not only alleviates oxidative stress and liver injury by inhibiting ROS but also negatively regulates lipid accumulation and inhibits the synthesis and release of proinflammatory mediators. Moreover, it has been proven to have significant protective effects in NASH mouse models, with these protective effects achieved through a combination of multiple mechanisms [32,33]. After in-depth investigation of the signaling pathways involved in hepatocyte lipid metabolism, it was found that OCA, by activating FXR, can significantly induce the expression of SHP, subsequently reducing the activity of key enzymes involved in fat production and decreasing hepatic lipid deposition [34,35]. Additionally, SHP exerts antioxidant effects by interfering with the uptake of fatty acids by hepatocytes, inhibiting lipid synthesis, and promoting lipid transport, further reducing lipid deposition in the liver, decreasing inflammatory responses, and improving NASH fibrosis [36]. Studies have shown that the activation of FXR can regulate lipid metabolism and improve the lipid status of hepatocytes by reducing the levels of inflammatory cytokines such as IL-15 and TNF-α [37]. This not only alleviates the inflammatory response in the liver but also affects other factors closely related to liver metabolism and immune responses [38,39].

**Fig. 6:**
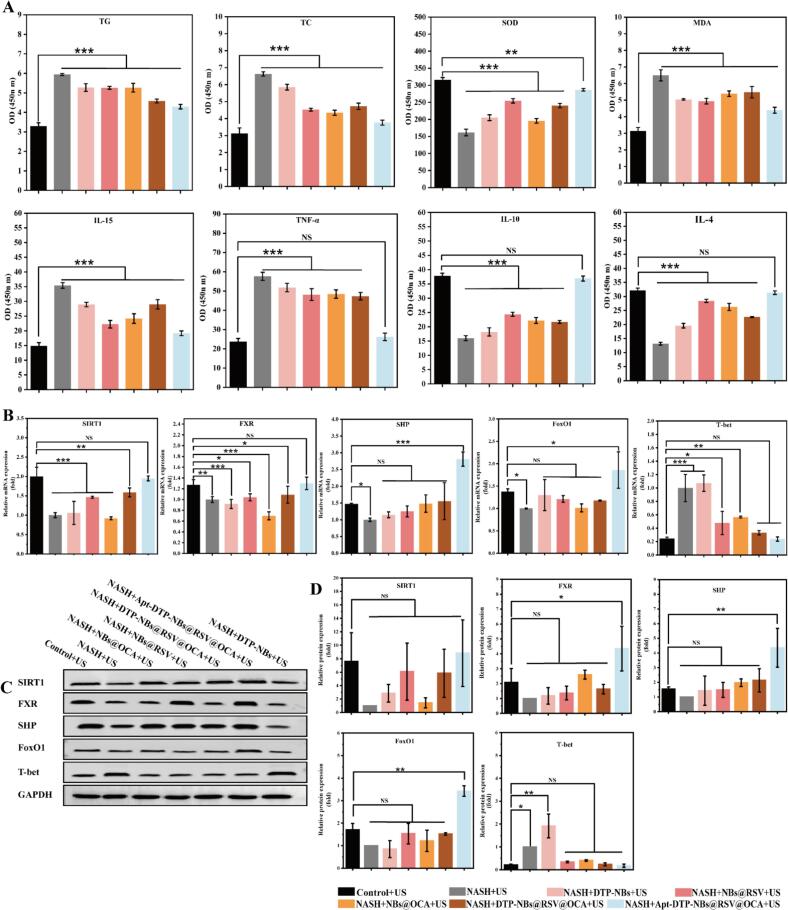
(A) Improvements in lipid metabolism, inflammation, and oxidative stress by Apt-DTP-NBs@RSV@OCA *in vitro*. Levels of TG, TC, SOD, MDA, IL-10, TNF-α, IL-15, and IL-4 measured via ELISA. (B) Molecular mechanism of Apt-DTP-NBs@RSV@OCA on NASH treatment. RT −PCR analysis of mRNA levels for SIRT1, FXR, SHP, FoxO1, and T-bet. (C) Western blotting showing protein expression of SIRT1, FXR, SHP, FoxO1, and T-bet. (D) Western blotting analysis of protein expression levels for SIRT1, FXR, SHP, FoxO1, and T-bet. Values shown are means ± SD, *p < 0.05, **p < 0.01, ***p < 0.001 indicate statistical significance, n = 3.

This study aimed to investigate the impact of Apt-DTP-NBs@RSV@OCA on biochemical parameters in NASH cells. ELISA kits were used to detect the levels of lipid metabolism (TG, TC), inflammatory cytokines(IL-4, IL-10, IL-15, TNF-α), and oxidative stress(SOD, MDA).Multiple groups were included: HepG2 + US (control), NASH + US (disease control), and various treatment groups.The treatment groups had different nanoparticle formulations and sizes. Ultrasound (US) stimulation was applied to all treatment groups. After 24 h of treatment, cellular enzyme expression was assessed. The results, illustrated in (Fig. 6A), indicate that the NASH + Apt-DTP@RSV@OCA@NBs + US group showed the lowest OD values for TG, TC, IL-15, TNF-α and MDA, with statistically significant differences compared to the other groups (*P* < 0.05). Conversely, the OD value for the anti-inflammatory cytokine IL-10, as well as for IL-4, was increased in the same group, showing statistically significant differences compared to the other groups (*P* < 0.05). These findings suggest that Apt-DTP@RSV@ OCA@NBs, particularly at the specified size and with US stimulation, may effectively improve lipid accumulation, inflammation, and oxidative stress in NASH cells.

#### 3.7.2 Real-time polymerase chain reaction (RT-PCR)

Notably, FoxO1 is closely associated with various cellular signaling molecules and metabolic pathways in liver metabolism and immune responses. After FXR activation, it regulates the expression of inflammatory cytokines, thereby upregulating the transcriptional activity of FoxO1, and exerts synergistic effects in liver metabolism and immune responses [22,40]. Research has also found that FoxO1 expression levels are higher in NASH patients compared to those with simple fatty liver disease, suggesting that FoxO1 may play an important role in the progression of NASH. This difference may represent an adaptive mechanism of the liver in response to persistent injury and inflammation [41].

Additionally, recent research has revealed the central role of the inflammatory cytokine IL-15 in the pathogenesis of NASH. The study found that IL-15 can induce the downregulation of FoxO1, which has a significant impact on the metabolic balance and immune homeostasis of the liver, leading to rapid upregulation of apoptosis-related factor ligands in CXCR6^+^ cells, triggering the release of T-box expressed in T cells (T-bet), exacerbating the nonspecific killing activity of CD8^+^ T cells, and ultimately resulting in hepatocyte damage and liver fibrosis [42]. Regulating the expression level of FoxO1 can affect the differentiation and effector functions of CD8^+^ T cells, with the release of T-bet being a critical link. Attenuating the nonspecific killing activity of CD8^+^ T cells may provide a potential intervention pathway for alleviating NASH. To further explore the exact roles of key factors such as FoxO1 and T-bet in the pathogenesis of NASH and their interregulatory relationships.

The results (Fig. 6B) indicated that in NASH cells, the mRNA expression levels of SHP and FoxO1 in the NASH + Apt-DTP-NBs@RSV@OCA + US group were significantly increased, with the differences being statistically significant (P < 0.05). Meanwhile, an elevation in the mRNA expression levels of SIRT1 and FXR was also observed in NASH cells, but no significant difference was detected when compared to the normal control group. In addition, a reduction in the mRNA expression level of T-bet was noted in NASH cells, but no significant difference was observed relative to the normal control group. This further underscores the pivotal role of the aptamer during the transfection process in enhancing the mRNA levels of SIRT1, FXR, SHP, and FoxO1. Concurrently, as the expression of SIRT1, FXR, SHP, and FoxO1 increased, the mRNA expression of T-bet decreased.

#### 3.7.3 Western blotting experiment

Additionally, Western Blotting was employed to measure the protein expression levels of FoxO1, SIRT1, FXR, SHP, and T-bet following the 48-hour transfection, as illustrated in (Fig. 6C). Notably, as shown in (Fig. 6D) that the T-bet protein expression level in the NASH + Apt-DTP-NBs@RSV@OCA + US group was observed to be the lowest, showing no significant difference compared to the control group. Additionally, the highest expression levels of SIRT1, FXR, SHP, and FoxO1 proteins were detected in this group, with the differences being statistically significant (*P* < 0.05). These results indicate that the T-bet level in NASH cells can be reduced to the level of normal cells by this nanocarrier. As expected, the protein-level analysis results were found to be consistent with the PCR results, confirming that SIRT1 and FXR were activated, FoxO1 and SHP were induced to overexpress, and the release of T-bet was suppressed by NASH + Apt-DTP-NBs@RSV@OCA + US.

Our experimental revealed that Apt-DTP-NBs@RSV@OCA activates the FXR and reduces ROS levels, thereby negatively regulating hepatic inflammatory cytokines. This, in turn, induces overexpression of the FoxO1 transcription factor and inhibits the release of T-bet, ultimately suppressing hepatocyte injury and liver fibrosis.

## 4 Conclusion

A US-driven, ROS-scavenging, and liver-targeted nanobubble system (Apt-DTP-NBs@RSV @OCA) co-encapsulating RSV and OCA has been successfully synthesized, providing a safe and efficient delivery platform for NASH therapy. Through cell characterization experiments, it was demonstrated that the synthesized nanobubbles exhibited excellent dispersion stability and suitable zeta potential size. Additionally, it was shown that at a drug concentration of 9.09 μg/mL, the nanobubbles displayed low cytotoxicity, and thus this concentration was selected for subsequent NASH cell therapy studies. Further research revealed that at this concentration, Apt-DTP@NBs@RSV@OCA not only accelerated drug absorption but also restored the cell state to a level comparable to that of the normal group. Through *in vitro* evaluations, it was demonstrated that Apt-DTP-NBs@RSV@OCA exhibited good targeting ability toward NASH cells and improved lipid metabolism. Meanwhile, *in vivo* studies in mice indicated that liver-targeted Apt-DTP-NBs@RSV@OCA significantly increased their effective concentration in the liver. In the NASH cell model, treatment with liver-targeted and ultrasound-driven Apt-DTP-NBs@RSV@OCA nanocarriers was found to significantly enhance cellular uptake of RSV and OCA and improve ROS scavenging capacity. Furthermore, in the NASH + Apt-DTP-NBs@RSV@OCA + US group, the levels of TG, TC, IL-15, TNF-α, and MDA were significantly downregulated, while the levels of IL-10, IL-4, and SOD were upregulated. Mechanistic studies revealed that the Apt-DTP-NBs@RSV@OCA + US group activated the FXR/SHP signaling pathway, enhanced FoxO1 activity, and inhibited T-bet release, thereby exerting therapeutic effects. These results suggest that this approach providing a therapeutic strategy for NASH.

In the future, the preparation process of the nanobubbles is expected to be further optimized, their application in various liver disease models to be explored, and preclinical as well as clinical studies to be conducted, which is anticipated to promote their practical application in the field of liver disease therapy.

## CRediT authorship contribution statement

**Jianru Lin:** Writing – original draft, Methodology, Funding acquisition, Data curation. **Jialin Chen:** Writing – review & editing, Methodology. **Mengdie Wang:** Methodology, Investigation, Data curation. **Kun He:** Software, Formal analysis. **CuiYan Lin:** Software, Formal analysis. **Xian Cao:** Supervision, Resources, Funding acquisition. **Jichuang Lai:** Resources. **Baohui Zeng:** Resources. **Xinmin Guo:** Writing – review & editing, Project administration, Investigation, Funding acquisition, Conceptualization.

## Funding

This work was supported by Science and Technology Projects in Guangzhou, China. (Grant Number: 2024A03J0676).

## Declaration of competing interest

The authors declare that they have no known competing financial interests or personal relationships that could have appeared to influence the work reported in this paper.
