# Supplementary material for: Ultrasound-driven ROS-scavenging nanobubbles for synergistic NASH treatment via FXR activation
Source: Ultrason Sonochem. 2025 Apr 26;118:107352. doi: 10.1016/j.ultsonch.2025.107352 (PMC12124726; doi:10.1016/j.ultsonch.2025.107352)
Supplement: Supplementary Data 1 [file mmc1.zip › Table_ULTSON 107352/Table_ULTSON 107352.docx]

**Supplementary Information**

**Table S1 Primer sequences used for quantitative real-time PCR analysis**

| Primer Name | Product Size (bp) | Primer Sequence (5' → 3') |
| --- | --- | --- |
| β-actin-qF-ck | 186 | F: TGGCACCCAGCACAATGAA |
|  |  | R: CTAAGTCATAGTCCGCCTAGAAGCA |
| NR1H4-qF1 | 237 | F: CGACAACAAAGTCATGCAGGGA |
|  |  | R: GGTCCAAAGTCTGAAATCCTGGTAG |

**Notes: F denotes forward primer, R denotes reverse primer. β-actin was used as an internal control for normalization.**
